# Complete genome sequence of *Thioalkalivibrio sp.* K90mix

**DOI:** 10.4056/sigs.2315092

**Published:** 2011-12-23

**Authors:** Gerard Muyzer, Dimitry Y. Sorokin, Konstantinos Mavromatis, Alla Lapidus, Brian Foster, Hui Sun, Natalia Ivanova, Amrita Pati, Patrik D'haeseleer, Tanja Woyke, Nikos C. Kyrpides

**Affiliations:** 1 Department of Biotechnology, Delft University of Technology, Delft, The Netherlands; 2 Department of Aquatic Microbiology, Institute for Biodiversity and Ecosystem Dynamics, University of Amsterdam, Amsterdam, The Netherlands; 3 Winogradsky Institute of Microbiology, Russian Academy of Sciences, Moscow, Russia; 4 Joint Genome Institute, Walnut Creek, California, USA; 5 Joint Bioenergy Institute, California, USA

**Keywords:** natronophilic, sulfide, thiosulfate, sulfur-oxidizing bacteria, soda lakes

## Abstract

*Thioalkalivibrio sp.* K90mix is an obligately chemolithoautotrophic, natronophilic sulfur-oxidizing bacterium (SOxB) belonging to the family *Ectothiorhodospiraceae* within the *Gammaproteobacteria*. The strain was isolated from a mixture of sediment samples obtained from different soda lakes located in the Kulunda Steppe (Altai, Russia) based on its extreme potassium carbonate tolerance as an enrichment method. Here we report the complete genome sequence of strain K90mix and its annotation. The genome was sequenced within the Joint Genome Institute Community Sequencing Program, because of its relevance to the sustainable removal of sulfide from wastewater and gas streams.

## Introduction

*Thioalkalivibrio sp.* K90mix is an obligately chemolithoautotrophic SOxB using CO_2_ as a carbon source and reduced inorganic sulfur compounds as an energy source. It belongs to the genus *Thioalkalivibrio*. This genus represents a dominant SOxB type in soda lakes – extremely alkaline and saline habitats - and is the first example of an obligate chemolithoautotroph capable of growing in saturated sodium carbonate brines. It forms a monophyletic group within the family *Ectothiorhodospiraceae* of the Gammaproteobacteria. The genus currently includes nine validly published species [1] and around 70, yet uncharacterized strains that are extremely salt-tolerant and genetically different from the characterized isolates recovered from hypersaline soda lakes [2-4]. The members are slow growing obligate autotrophs, well adapted to hypersaline (up to salt saturation) and alkaline (up to pH 10.5) conditions. Members of the genus *Thioalkalivibrio* have versatile metabolic capabilities, including oxidation of reduced sulfur compounds [1,2], denitrification [5,6] and thiocyanate utilization [7,8].

Apart from playing an important role in the sulfur cycle of soda lakes, *Thioalkalivibrio* species also are being used for the sustainable removal of sulfide from wastewater and gas streams [9,10]. In this process hydrogen sulfide is absorbed to a high salt alkaline solution, which is subsequently transferred to a bioreactor in which *Thioalkalivibrio* spp. oxidize HS^-^ to elemental sulfur. The produced biosulfur can then be used as a fertilizer or fungicide [9].

To get a comprehensive understanding of the molecular mechanism by which *Thioalkalivibrio sp.* K90mix oxidize sulfur compounds and adapts to extreme alkaline (up to pH 10.5) and hypersaline conditions (up to 4 M of Na^+^ or 3.6 M of K^+^) it is necessary to identify the genes that are involved in these adaptations. The most important issues in this are the mechanism of sulfide oxidation, carbon assimilation at high pH, and bioenergetic adaptation to high salt and high pH. Here we present a summary classification and a set of features for *Thioalkalivibrio sp.* K90mix together with the description of the genomic sequencing and annotation.

## Classification and features

Because of limited solubility of sodium carbonates in the biodesulfurization process, we made a series of enrichment cultures with an increasing ratio of potassium to sodium carbonate (potassium carbonates have a 2-5 times higher solubility than sodium carbonates). *Thioalkalivibrio sp.* K90mix was isolated from a culture that was inoculated with a mixture of sediment samples from different hypersaline soda lakes and was grown at the maximal possible substitution of sodium for potassium, 3.6 M K^+^/0.4 M Na^+^ (90% substitution).

**Figure 1:**
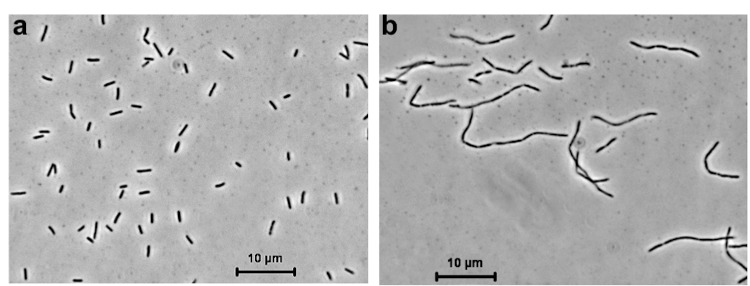
Phase contrast micrographs of the cell morphology of *Thioalkalivibrio sp.* K90mix grown at pH 10 and 4 M Na^+^ **(a),** or 3.6 M K^+^/0.4 M Na^+^ **(b).**

**Figure 2:**
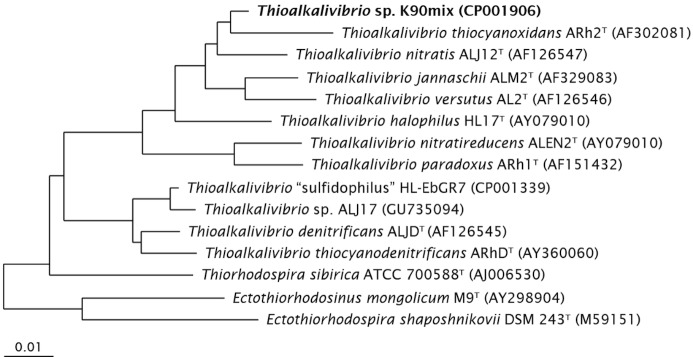
Phylogenetic tree based on 16S rRNA sequences showing the phylogenetic position of *Thioalkalivibrio sp.* K90mix. The sequence was aligned to sequences stored in the SILVA database using the SINA Webaligner [24]. Subsequently, the aligned sequences were imported into ARB [25], and a neighbor joining tree was constructed. Sequences of members from the *Alphaproteobacteria* were used as outgroup, but were pruned from the tree. The scale bar indicates 1% sequence difference.

*Thioalkalivibrio sp.* K90mix has rod-shaped cells with a polar flagellum (Figure 1), that elongate at high concentrations of K^+^ (Figure 1b). The strain is obligately alkaliphilic with a pH optimum of 10 (Table 1). It can tolerate a salinity of 4.0 M total Na^+^, but has an optimum of 0.3 M, sulfide concentrations up to 1 mM and a temperature up to 40°C. It has a preference for carbonate and sulfate as counter-anions over chloride and, therefore must be called “natronophilic”, instead of “haloalkaliphilic”. It utilizes ammonia, nitrate and nitrite as a nitrogen source. On the basis of 16S rRNA gene sequencing the strain belongs to the genus *Thioalkalivibrio* within the *Gammaproteobacteria* with *T. thiocyanoxidans* and *T. nitratis* as the closest described species (Figure 2). Most of the yet undescribed *Thioalkalivibrio* isolates from hypersaline lakes of Siberia and Mongolia also belong to this core genetic cluster of the genus *Thioalkalivibrio*.

**Table 1:** Classification and general features of *Thioalkalivibrio sp.* K90mix according to the MIGS recommendations [11].

**MIGS ID**	**Property**	**Term**	**Evidence code**
	Current classification	Domain *Bacteria*	TAS [12]
		Phylum *Proteobacteria*	TAS [13]
		Class Gammaproteobacteria	TAS [14,15]
		Order *Chromatiales*	TAS [16]
		Family *Ectothiorhodospiraceae*	TAS [17]
		Genus *Thioalkalivibrio*	TAS [18-21]
		Species *Thioalkalivibrio sp.* K90mix	NAS
	Gram stain	negative	TAS [2]
	Cell shape	rod-shaped	TAS [2]
	Motility	motile	TAS [2]
	Sporulation	non-sporulating	TAS [2]
	Temperature range	Mesophile; maximum at 41^o^C	TAS [2]
	Optimum temperature	34^o^C	TAS [2]
MIGS-6.3	Salinity range	0.2-4.0 M Na^+^ (opt. 0.3 M)	TAS [2]
MIGS-22	Oxygen requirement	aerobic	TAS [2]
	Carbon source	HCO_3_^-^	TAS [2]
	Energy source	Sulfide/polysulfide, thiosulfate, sulfur, sulfite	TAS [2]
MIGS-6	Habitat	soda lakes	TAS [2]
MIGS-15	Biotic relationship	free-living	TAS [2]
MIGS-14	Pathogenicity	none	NAS
	Biosafety level	1	TAS [22]
	Isolation	Soda lake sediments	TAS [4]
MIGS-4	Geographic location	Kulunda Steppe, Altai, Russia	TAS [4]
MIGS-5	Sample collection time	2005	NAS
MIGS-4.1	Latitude	51.41	TAS [23]
MIGS-4.2	Longitude	79.48	TAS [23]
MIGS-4.3	Depth	Not applicable	
MIGS-4.4	Altitude	Sea level	NAS

Evidence codes - IDA: Inferred from Direct Assay (first time in publication); TAS: Traceable Author Statement (i.e., a direct report exists in the literature); NAS: Non-traceable Author Statement (i.e., not directly observed for the living, isolated sample, but based on a generally accepted property for the species, or anecdotal evidence). These evidence codes are from of the Gene Ontology project. If the evidence code is IDA, then the property was directly observed by one of the authors or an expert mentioned in the acknowledgements.

## Genome sequencing information

### Genome project history

Strain K90mix was selected for sequencing in the 2007 Joint Genome Institute Community Sequencing Program, because of its relevance to bioremediation. A summary of the project information is presented in Table 2. The complete genome sequence was finished in February 2010. The GenBank accession numbers are NC_013889 and NC_013930 for the chromosome and plasmid, respectively. The genome project is listed in the Genome OnLine Database (GOLD) [26] as project Gc01217. Sequencing was carried out at the Joint Genome Institute (JGI) Finishing was done by JGI-Los Alamos National Laboratory (LANL) and initial automatic annotation by JGI-Oak Ridge National Laboratory (ORNL).

**Table 2:** Genome sequencing project information

**MIGS ID**	**Characteristic**	**Details**
MIGS-28	Libraries used	6kb and 40kb Sanger and 454 standard libraries
MIGS-29	Sequencing platform	ABI-3730, 454 GS FLX Titanium
MIGS-31.2	Sequencing coverage	10.0× Sanger, 32.1× pyrosequence
MIGS-31	Finishing quality	Finished
	Sequencing quality	Less than one error per 100 kb
MIGS-30	Assembler	Newbler, PGA
MIGS-32	Gene calling method	Prodigal, GenePRIMP
	GenBank ID	NC_013889
	GenBank date of release	February 21, 2010
	GOLD ID	Gc01217
	NCBI project ID	30759
	IMG Taxon ID	646564584
MIGS-13	Source material identifier	Personal culture collection, Winogradsky Institute of Microbiology, Moscow
	Project relevance	Bioremediation

### Growth conditions and DNA isolation

*Thioalkalivibrio sp.* K90mix was grown with 40 mM thiosulfate as an energy source in standard sodium carbonate-bicarbonate medium at pH 10 and 2 M Na^+^ [2] at 35^o^C with shaking at 200 rpm. The cells were harvested by centrifugation and stored at minus 80°C for DNA extraction. Genomic DNA was obtained using phenol-chloroform-isoamylalcohol (PCI) extraction. The genomic DNA was extracted using PCI and precipitated with ethanol. The pellet was dried under vacuum and subsequently dissolved in water. The quality and quantity of the extracted DNA was evaluated using the DNA Mass Standard Kit provided by the JGI.

### Genome sequencing and assembly

The genome of *Thioalkalivibrio sp.* K90mix was sequenced using a combination of Sanger and 454 sequencing platforms. All general aspects of library construction and sequencing can be found at the JGI website [27]. Pyrosequencing reads were assembled using the Newbler assembler version 1.1.02.15 (Roche). Large Newbler contigs were broken into 3,292 overlapping fragments of 1,000 bp and entered into assembly as pseudo-reads. The sequences were assigned quality scores based on Newbler consensus q-scores with modifications to account for overlap redundancy and adjust inflated q-scores. A hybrid 454/Sanger assembly was made using the PGA assembler. Possible mis-assemblies were corrected and gaps between contigs were closed by editing in Consed, by custom primer walks from sub-clones or PCR products. A total of 181 Sanger finishing reads were produced to close gaps, to resolve repetitive regions, and to raise the quality of the finished sequence. Illumina reads were used to improve the final consensus quality using an in-house developed tool (the 'Polisher' [28]). The error rate of the completed genome sequence is less than 1 in 100,000. Together, the combination of the Sanger and 454 sequencing platforms provided 42.1× coverage of the genome. The final assembly contains 28,443 Sanger reads (10.0×) and 419,015 pyrosequencing reads (32.1×).

### Genome annotation

Genes were identified using Prodigal [29] as part of the Oak Ridge National Laboratory genome annotation pipeline followed by a round of manual curation using the JGI GenePRIMP pipeline [30]. The predicted CDSs were translated and used to search the National Center for Biotechnology Information (NCBI) nonredundant database, UniProt, TIGRFam, Pfam, PRIAM, KEGG, COG, and InterPro, databases. Additional gene prediction analysis and functional annotation was performed within the Integrated Microbial Genomes Expert Review (IMG-ER) platform [31].

## Genome properties

**Figure 3:**
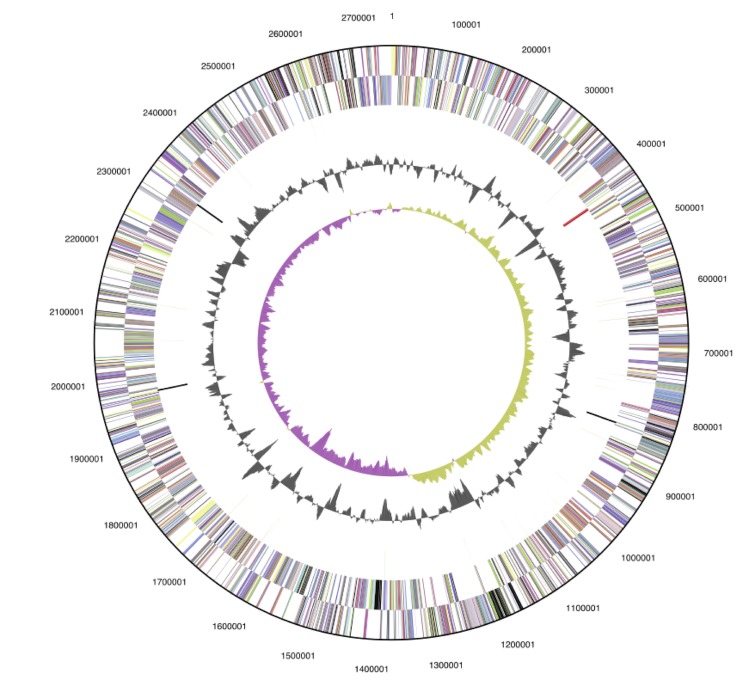
Graphical circular map of the chromosome of *Thioalkalivibrio sp.* strain K90mix. From outside to the center: Genes on the forward strand (Colored by COG categories), Genes on the reverse strand (colored by COG categories), RNA genes (tRNAs green, rRNAs red, other RNAs black), GC content, GC skew.

The genome of strain K90mix consists of a circular chromosome with a size of 2.74 Mbp (Figure 3) and a linear plasmid of 240 Kbp. The G+C percentage determined from the genome sequence is 65.54%, which is similar to the value determined by thermal denaturation (65.8±0.5 mol%). There are 2942 genes of which 2888 are protein-coding genes and the remaining 54 are RNA genes. 33 pseudogenes were identified, constituting 1.12% of the total number of genes. The genome is smaller than that of “Thioalkalivibrio sulfidophilus” HL-EbGr7 [32], 2.98 Mbp versus 3.46 Mbp, but has a similar percentage of protein-coding genes (98.16% versus 98.06%). The properties and statistics of the genome are summarized in Table 3, and genes belonging to COG functional categories are listed in Table 4.

**Table 3:** Genome statistics

**Attribute**	**Value**	**% of Total**
Genome size (bp)	2,985,056	100.00%
DNA coding region (bp)	2,673,521	89.56%
DNA G+C content (bp)	1,956,383	65.54%
Number of replicons	1	
Extrachromosomal elements (plasmid)	1	
Total genes	2,942	100.00%
RNA genes	54	1.84%
rRNA operons	3	0.10%
Protein-coding genes	2,888	98.16%
Pseudogenes	33	1.12%
Genes in paralog clusters	267	9.08%
Genes assigned to COGs	2,139	72.71%
Genes assigned Pfam domains	2,292	77.91%
Genes with signal peptides	934	31.75%
CRIPR repeats	2	

**Table 4:** Number of genes associated with the general COG functional categories.

**Code**	**Value**	**%age**	**Description**
J	156	6.57	Translation, ribosomal structure and biogenesis
A	1	.04	RNA processing and modification
K	86	3.62	Transcription
L	164	6.91	Replication, recombination and repair
B	2	0.08	Chromatin structure and dynamics
D	41	1.73	Cell cycle control, mitosis and meiosis
Y	0	0.0	Nuclear structure
V	35	1.47	Defense mechanisms
T	166	6.99	Signal transduction mechanisms
M	176	7.41	Cell wall/membrane biogenesis
N	94	3.96	Cell motility
Z	0	0.0	Cytoskeleton
W	0	0.0	Extracellular structures
U	97	4.09	Intracellular trafficking and secretion
O	110	4.63	Posttranslational modification, protein turnover, chaperones
C	146	6.15	Energy production and conversion
G	79	3.33	Carbohydrate transport and metabolism
E	155	6.53	Amino acid transport and metabolism
F	66	2.78	Nucleotide transport and metabolism
H	146	6.15	Coenzyme transport and metabolism
I	65	2.74	Lipid transport and metabolism
P	119	5.01	Inorganic ion transport and metabolism
Q	42	1.77	Secondary metabolites biosynthesis, transport and catabolism
R	240	10.11	General function prediction only
S	188	7.92	Function unknown
-	803	27.29	Not in COGs

## Insights from the genome sequence

### Autotrophic growth

**Figure 4:**
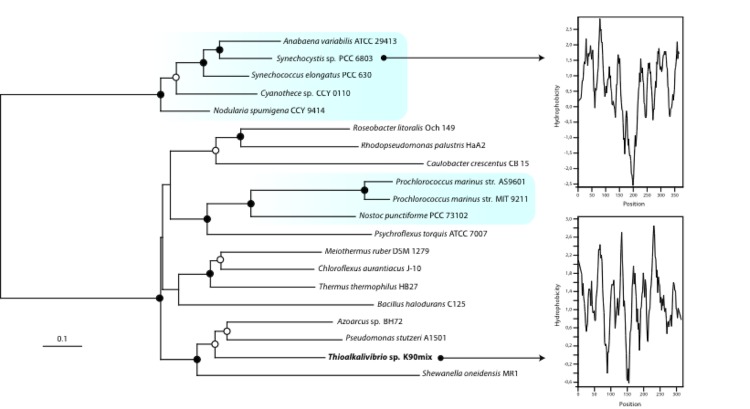
Phylogeny and hydropathy of StbA from different microorganisms. The blue boxes indicate sequences belonging to the cyanobacteria. The dots on the branches are bootstrap values between 50% and 75% (open dots) or between 75% and 100% (closed dots) The scale bar indicated 10% sequence difference. The sequence of *Thioalkalivibrio sp.* K90mix is indicated in bold. Hydropathy profiles were made for the StbA protein sequences of *Synechocystis* sp. PCC6803 and *Thioalkalivibrio sp.* K90mix to indicate transmembrane spanning domains.

As mentioned before, [32] autotrophic growth at extremely high pH is a problem, because inorganic carbon is mainly present as carbonate (with bicarbonate as a minor fraction) at pH values above 10. This would demand active transport of bicarbonate into the cell. We found a gene related to stbA encoding a Na^+^/HCO_3_^-^ symporter in the marine cyanobacterium *Synechocystis* sp. strain PCC 6803 [33]. Figure 4 shows a phylogenetic tree of different sequences related to StbA and the hydrophobicity profiles of StbA of *Synechocystis* sp. PCC 6803 and *Thioalkalivibrio* sp. K90mix. In addition, we have found genes for the large (TK90_0858) and small subunit (TK90_0859) of RuBisCO form 1Ac, and for the synthesis of α-carboxysomes (TK90_0860 – TK90_0866), including *csoSCA* encoding a carboxysome shell alpha-type carbonic anhydrase, which was also found in genomic analysis of “*Thioalkalivibrio sulfidophilus”* HL-EbGr7 [32].

### Sulfur metabolism

**Figure 5:**
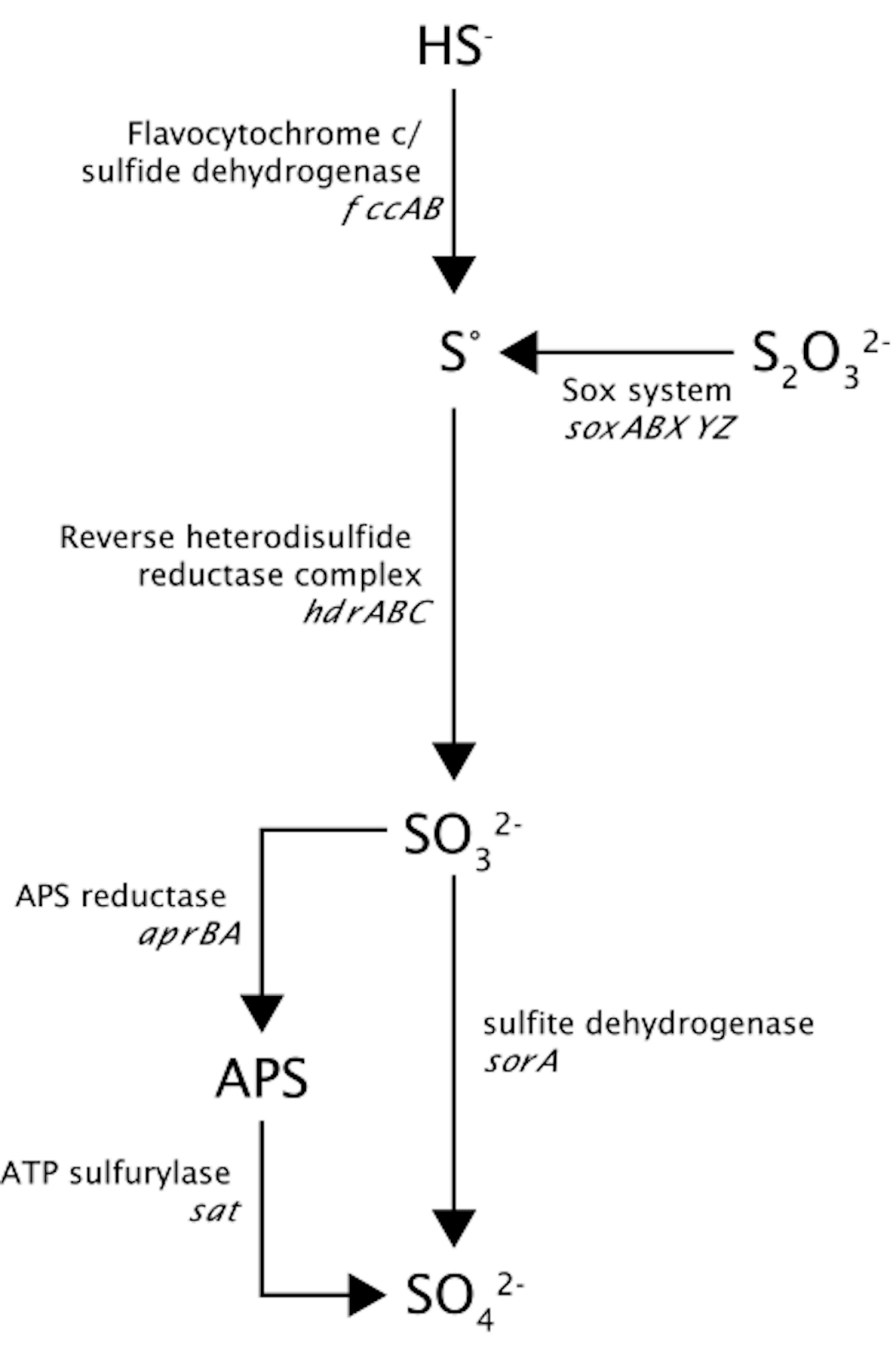
Schematic overview of the different genes that are involved in the oxidation of sulfur compounds, although the role of the Hdr complex has not been proven yet. The genes encoding the reverse dissimilatory sulfite reductase (dsr), which are present in the genome of *Thioalkalivibrio sulfidophilus*, are absent in the genome of *Thioalkalivibrio sp.* K90mix.

**Figure 6:**
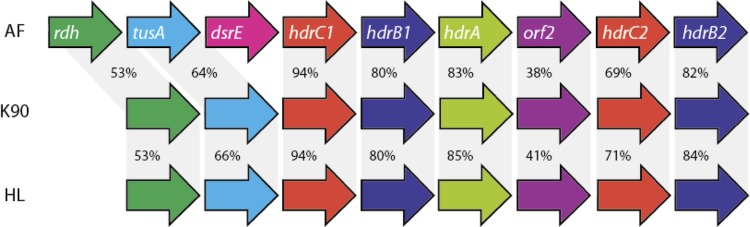
Comparison of the hdr cluster of *A. ferroooxidans* ATCC 23270 (AF), *Thioalkalivibrio sp.* K90mix (K90) and *Thioalkalivibrio sulfidophilus* HL-EbGR7 (HL). The heterodisulfide reductase complex consists of the genes encoding HdrC1, HdrB1, HdrA, orf2, HdrC2 and HdrB2. The accessory proteins are Rhd, and TusA. DsrE was not found in this order, but was present at other places in the genome. The percentage of amino-acid similarity is indicated.

*Thioalkalivibrio sp.* K90mix can oxidize sulfide/polysulfide, thiosulfate, sulfite (*in vitro*) and elemental sulfur to sulfate. Elemental sulfur is formed as an intermediate during sulfide and thiosulfate oxidation at oxygen limitation and near-neutral pH. Figure 5 shows a schematic overview of the different genes that are involved in the oxidation of sulfur compounds. The genome of *Thioalkalivibrio sp.* K90mix contains genes for flavocytochrome *c*/sulfide dehydrogenase (TK90_0236), which oxidizes sulfide to elemental sulfur. It contains an incomplete set of sox genes including *soxYZ* (TK90_0123 and TK90_0124), *soxAX* (TK90_0432 and TK90_0433) and two copies of *soxB* (TK90_0627 and TK90_1150), but is lacking *soxCD,* which would allow oxidizing the sulfane atom of thiosulfate to the state of elemental sulfur, but no further. However, it does not contain the reverse dissimilatory sulfite reduction pathway to oxidize sulfur to sulfite, which has been found in the genome of “*Thioalkalivibrio sulfidophilus”* HLEbGr7 [32]. Absence of dsr genes has also been found for the green sulfur bacterium *Chloroherpeton thalassium* that can oxidize sulfide to elemental sulfur, but subsequently can only oxidize the produced sulfur very slowly [34], probably due to the absence of dsr. Frigaard and Dahl [35] suggested that the presence of a RuBisCo-like protein (RLP) might be involved in sulfur oxidation [36]. Genes encoding for the RuBisCo-like protein were not found, nor were genes encoding sulfur dioxygenase or sulfur oxygenase-reductase, which can oxidize or disproportionate sulfur in several acidophilic bacteria and archaea [37]. However, we found a gene cluster encoding two sulfur transferases (*rhd,* TK90_0630; *sirA,* TK90_0631) and a heterodisulfide reductase complex (TK90_0632 - TK90_0637) consisting of *hdrA, hdrB,* and *hdrC* (Figure 6). *dsrE* was missing in this cascade, but was present at 3 other places in the genome (TK_0511, TK_0639, TK90_1244).

The Hdr complex plays a function in the energy metabolism of methanogens [38] and sulfate-reducing prokaryotes [39]. In methanogens, the enzyme complex catalyzes the reversible reduction of the disulfide (CoM-S-S-CoB) of the two methanogenic thiol-coenzymes, coenzyme M (CoM-SH) and coenzyme B (CoB-SH); in sulfate reducing microorganisms the substrate (X-S-S-X) is not known. Recently, the genes encoding the Hdr complex have also been detected in the genomes of the acidophilic sulfur oxidizing bacteria *Acidithiobacillus ferrooxidans* [40] and *Acidithiobacillus caldus* [41]. Quantrini and co-workers [40] hypothesized that Hdr, like the dissimilatory sulfite reductase (dsr), is working in reverse, whereby sulfur (i.e., sulfane atom) is oxidized to sulfite, and electrons are donated to the quinone pool. Although the role of the Hdr complex in the sulfur metabolism still has to be confirmed by expression analysis, Hdr genes have not yet been detected in the genomes of the neutrophilic chemolithotrophic sulfur-oxidizing bacteria *Thiomicrospira crunogena* and *Thiobacillus denitrificans* or of phototrophic sulfur-oxidizing bacteria *Allochromatium vinosum* and *Halorhodospira halophila*. The absence of a reverse dsr pathway in *Thioalkalivibrio sp.* K90mix might be the reason why it can only tolerate sulfide concentrations up to 1 mM. Sulfite can be oxidized further to sulfate, either directly by sulfite dehydrogenase (*sorA*; TK90_0686) or indirectly via adenosine-5’-phosphosulfate (APS) by APS reductase encoded by *aprBA* (TK90_0064 and TK90_0065) and ATP sulfurylase encoded by *sat* (TK90_0062) [42]. All these genes are present in the investigated genome.

### Energy metabolism and pH homeostasis

At this time, it is not clear how *Thioalkalivibrio sp.* K90mix can withstand the harsh conditions of high pH and salinity. The difference between the pH of the environment (pH 10) and the pH in the cell (pH 8) causes a reversed ΔpH and consequently lowers the proton motive force (PMF). Therefore, *Thioalkalivibrio* requires a special molecular mechanisms to obtain enough energy for growth. It certainly needs this energy, because the production of osmolytes, to withstand the high concentrations of salts, costs 55 molecules of ATP for one molecule of glycine betaine, and 110 molecules of ATP for 1 molecule of sucrose [43]. In addition, the chemolithoautotrophic life style of CO_2_ fixation is energetically very expensive. The redox potential of the substrate couple S°/HS- (-260 mV) is more positive that the potential of NAD^+^/NADH (-340 mV) and therefore the direct reduction of NAD^+^ in order to supply reducing equivalents for CO_2_ fixation is not possible. Reverse electron transport is necessary in order to produce enough NADH, necessary for CO_2_ fixation, which costs extra energy. In addition, because of the large pH gradient over the cell membrane *Thioalkalivibrio* needs special mechanisms to keep the intracellular pH around neutral (pH homeostasis), which again is an energy requiring process.

The genome has revealed genes encoding similar proteins as those found for “*Thioalkalivibrio sulfidophilus”* HL-EbGr7 [32]. We found genes for a proton-driven F0F1-type ATP synthase (i.e., subunit A TK90_2593, B TK90_2591, and C TK90_2592of the F0 subcomplex, and subunit alpha (TK90_2589), beta (TK90_2587), gamma (TK90_2588), delta (TK90_2590), and epsilon (TK90_2586) of the F1 subcomplex), genes encoding the proton-translocating NADH dehydrogenase (*nuoABCDEFGHIJKLMN*) (TK90_0708 to TK90_0721), as well as the genes for a putative primary sodium pump Rnf [44] (*rnfABCDGE*) (TK90_1790 to TK90_1795). In addition, we found several genes encoding different secondary sodium-dependent pumps, such as the Na^+^/H^+^ antiporters NhaP (TK90_1831) and Mrp (*mnhA-G*) (TK90_0748 to TK90_0752), which according to Padan et al. [45] both play an essential role in alkaline pH homeostasis. In addition, we found genes encoding transporters belonging to the SulP family (TK90_0019, TK90_0897, TK90_0985). Transporters of this group could be involved in the low affinity, but high flux of bicarbonate uptake [46]. In addition, genes encoding the sodium-depending flagellar motor PomA/B (TK90_1180 and TK90_1181) are also present in the genome (see below for more details). As *Thioalkalivibrio sp.* K90mix can stand high concentrations of potassium, we also searched for K^+^-transporters and found genes encoding the following transporters: TrkA-C (TK90_0502), TrkA-N (TK90_2266) and TrkH (TK90_2267) that are part of the potassium uptake system [47].

### Chemotaxis and motility

We found different genes encoding methyl-accepting chemotaxis sensory transducers (TK90_0580, TK90_0949, TK90_1402, TK90_2562, TK90_2397) that are involved in chemotaxis. One of these genes, *Aer* (TK90_0580), encodes a redox sensor involved in aerotaxis. In *E. coli*, Aer regulates the motility behavior in gradients of oxygen, redox potential and certain nutrients by interacting with the CheA-CheW complex. We found genes encoding several different proteins of this complex, CheA (TK90_1178), CheW (TK90_1183 and TK90_1184), CheY (TK90_1176), CheZ (TK90_1177), CheB (TK90_1179), CheV (TK90_0924) and CheR (TK90_0925). Chemotaxis consists of a complex cascade of different reactions: the redox sensor Aer senses a difference in redox potential induced by a change in the environmental oxygen concentration, which leads to the autophosphorylation of the histidine protein kinase CheA. CheA phosphorylates CheY, which will switch on the flagellar motor (see [48] for a detailed overview). CheW acts as an adaptor protein, while CheB, CheR, CheZ, and CheV are involved in feedback regulation.

**Figure 7:**
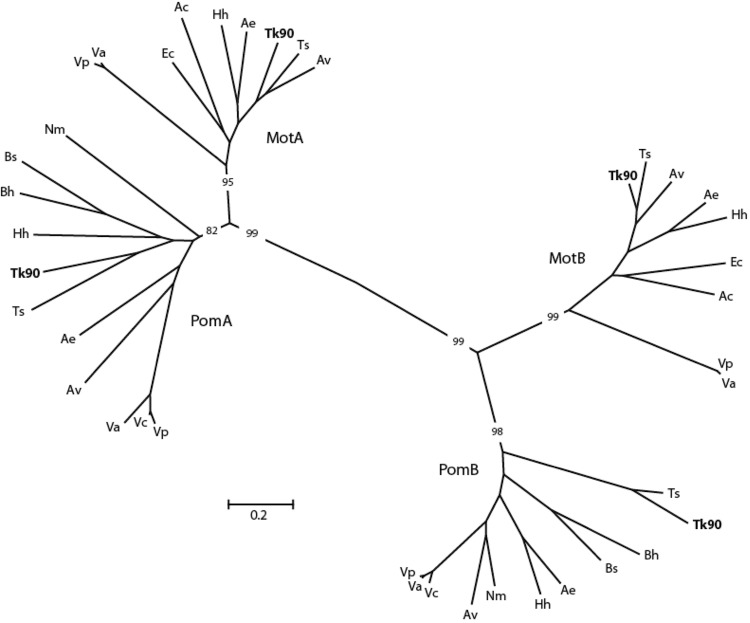
Phylogenetic tree based on protein sequences of different flagellar motors. The sequences of the proton-driven flagellar motor MotAB from *E. coli* (ECDH10B_2031, ECDH10B_2030) and of the sodium-driven flagellar motor PomAB from *V. cholerae* (VC0892, VC0893) were used as reference proteins. Other proteins were selected after BLAST analysis. Subsequently, the selected protein sequences were aligned used Clustal W, and a neighbor joining tree was drawn using MEGA 5. Ac, *Acidithiobacillus caldus* ATCC 51756; Ae, *Alkalilimnicola* *ehrlichei* MLHE-1; Av, *Allochromatium vinosum* DSM 180; Bh, *Bacillus halodurans* C125; Bs, *Bacillus subtilis* strain 168; Ec, *Escherichia coli* DH10B; Hh, *Halorhodospira* *halophila* SL1; Nm, *Nitrococcus mobilis* NB-231; Ts, *Thioalkalivibrio sulfidophilus* HLEbGR7; Tv-K90, *Thioalkalivibrio sp.* K90mix; Va, *Vibrio alginolyticus* 12G01; Vc, *Vibrio* *cholera* O1; Vp, *Vibrio parahaemolyticus* RIMD2210633. The sequences of *Thioalkalivibrio sp.* K90mix are shown in bold type. The bar indicates 20% sequence difference. Numbers on the branches indicate percentage bootstrap values from 1000 iterations; only those values are shown that distinguish the different flagellar motors. The CheW sequence of *Thioalkalivibrio sp.* K90mix (TK90_1183) was used as an outgroup, but was pruned from the tree.

*Thioalkalivibrio sp.* K90mix has all the genes that are indispensable for the production of flagellar proteins (FlgA, TK90_0923; FlgB, TK90_0926; FlgC, TK90_0927; FlgD, TK90_0928; FlgE, TK90_0929; FlgF, TK90_0930; FlgG, TK90_0931; FlgH, TK90_0932; FlgI, TK90_0933; FlgK, TK90_0935; FlgL, TK90_0936; FlgM, TK90_0922; FlgN, TK90_0921; FliC, TK90_1400, 1448, 1450; FliD, TK90_1447; FliE, TK90_1157; FliF, TK90_1158; FliG, TK90_1159; FliH, TK90_1160; FliI, TK90_1161; FliJ, TK90_1162; FliK, TK90_1163; FliM, TK90_1165; FliN, TK90_1166; FliO, TK90_1167; FliP, TK90_1168; FliQ, TK90_1169; FliR, TK90_1170; FliS, TK90_1446; FlhA, TK90_1172; FlhB, TK90_1171; see Table 1 in [49]). Phylogenetic analysis of sequences encoding different flagellar motors showed, with significant bootstrap values, genes encoding both proton-driven motors (TK90_0578, TK90_0577) related to *E. coli* MotA and MotB, as well as sodium-driven motors (TK90_1180, TK1181) related to *Vibrio* *cholera* PomA and PomB and *Bacillus subtilis* MotP and MotS (Figure 7). The results also show the presence of proton- and sodium-driven flagellar motors in the *Ectothiorhodospiraceae*, “*Thioalkalivibrio sulfidophilus”*, *Halorhodospira halophila*, and *Alkalilimnicola* *ehrlichei*, as well as in *Achromatium* *vinosum,* *Acidithiobacillus caldus*, *Vibrio alginolyticus* and *Vibrio parahaemolyticus*. *Vibrio cholerae*, *Bacillus subtilis*, *B. halodurans*, and *Nitrococcus mobilis* only have sodium-driven flagellar motors. Grouping of the proton-driven MotA and sodium-driven PomA has also been found by Krulwich et al. [50].

### Transposases and environmental stress

Comparative analysis of the genomes of “*Thioalkalivibrio* *sulfidophilus*” HL-EbGr7 [32] and *Thioalkalivibrio sp.* K90mix showed a greater abundance of genes encoding different transposases (i.e., COG2801, COG3328, COG3547) in the latter. Transposases are enzymes that can move specific sequences of DNA, known as transposons or transposable elements, within the genome. Krulwich [51] found that the genome of the alkaliphilic bacterium *Bacillus halodurans* C125 contained 112 transposase genes as compared to 10 in the genome of its closest non-alkaliphilic relative *B. subtilis*. She suggested that this might be one of the mechanisms of alkaliphilic adaptation at the genome level. Although strains HL-EbGr7 and K90mix are both obligately alkaliphilic, they differ in salt tolerance. HL-EbGr7 can tolerate only low (up to 1.5 M) salt concentrations, while K90mix can tolerate high (up to 4 M) salinities.

Capy et al. [52] mentioned that environmental stress might stimulate transposition and consequently increase the genetic variability, which can be beneficial for the adaptation to novel environmental conditions. Foti et al. [4] used rep-PCR [53] to study the genetic diversity within the genus *Thioalkalivibrio* and found a relatively high diversity of 56 genotypes among 85 strains that were isolated from different soda lakes in Africa and Asia. In addition, preliminary enrichment experiments with potassium carbonate instead of sodium carbonate and higher concentrations of chloride selected populations of high salt-tolerant *Thioalkalivibrio* strains with different rep-PCR patterns (unpublished results), which might be an indication that transposition might occur more frequently in strains with a wide range of salt tolerance.

### Oxidative stress

Reactive oxygen species (ROS), such as superoxides (O_2_^-^) and hydrogen peroxidase (H_2_O_2_), are naturally produced at hypersaline conditions and are deleterious to cellular macromolecules. To protect themselves from this oxidative stress, *Thioalkalivibrio sp.* K90mix and “*Thioalkalivibrio* s*ulfidophilus”* HL-EbGr7 have several defense mechanisms. Superoxides are converted to oxygen and hydrogen peroxide by the enzyme superoxide dismutase (TK90_0947, Tgr7_2463), while hydrogen peroxide is converted to oxygen by hydroperoxidase (TK90_0947, Tgr7_1107) or to H_2_O by the cytochrome C peroxidase (TK90_0812, Tgr7_2739). In addition, *Thioalkalivibrio sp.* K90mix produces high concentrations of a specific membrane-bound yellow pigment named ‘natronochrome’ [54]. The pigment has a high degree of unsaturation and might also play a role in the protection against reactive oxygen species (ROS). The gene(s) responsible for the synthesis of this anti-oxidant remains to be identified.

### Osmotic stress

*Thioalkalivibrio sp.* K90mix is an extremely salt-tolerant bacterium. It can grow in saturated sodium and potassium carbonate and sodium sulfate brines containing up to 4 M Na^+^/K^+^ but, in contrast to halo-alkaliphiles, it is inhibited by high concentrations of chloride. So, a more proper term for such an extremophile would be an “extreme natronophile”. To withstand these extreme salinities, it synthesizes glycine betaine as the main compatible solute; the genome contains the genes for glycine sarcosine N-methyltransferase (TK90_0179) and sarcosine dimethylglycine methyltransferase (TK90_0180). In addition, the genome contains the gene for sucrose phosphate synthase (TK90_2312) to produce sucrose as a compatible solute. Genes for ectoine were not found.
